# Importancia del cateterismo de senos petrosos inferiores en el diagnóstico de síndrome de Cushing, a propósito de un caso

**DOI:** 10.1515/almed-2022-0081

**Published:** 2022-11-25

**Authors:** Clara Jiménez García, Paula Sirera Sirera, María Eugenia Torregrosa Quesada, Victoria González Bueno, Rocío Alfayate Guerra

**Affiliations:** Laboratorio de Análisis Clínicos, Hospital General Universitario de Alicante Alicante, España; Unidad de Hormonas, Hospital General Universitario de Alicante Alicante, España

**Keywords:** cateterismo, test de estimulación con corticoliberina (CRH), síndrome de Cushing

## Abstract

**Objetivos:**

El diagnóstico del síndrome de Cushing (SC) sigue siendo un reto ya que sus síntomas y signos clínicos se superponen a los de otras enfermedades.

**Caso clínico:**

Varón remitido a Endocrinología por sospecha de SC. Para el estudio del SC se realizaron pruebas de cribado que confirman el hipercortisolimo y pruebas de diagnóstico que confirman un SC-adrenocorticotropina (ACTH) dependiente. Para discriminar entre origen hipofisario y ectópico se realizó el test de estimulación con corticoliberina (CRH). Ante la falta de respuesta en este test y la resonancia magnética nuclear (RMN) negativa se realizó el CSPI que sugería origen hipofisario por lo que se realizó cirugía transesfenoidal. El resultado anatomopatológico confirmó el origen corticotropo del tumor.

**Conclusiones:**

La investigación etiológica del SC y su diagnóstico diferencial constituye un proceso complejo utilizando diversas pruebas bioquímicas y de imagen. Es importante establecer la secuencia del estudio bioquímico realizando pruebas de cribado, confirmación y localización para establecer origen adrenal, hipofisario o ectópico. A pesar del buen rendimiento diagnóstico, este no es definitivo, especialmente en los casos ACTH dependiente. En nuestro caso, el resultado no concluyente en el test de CRH, requirió un procedimiento invasivo (CSPI) que resultó eficiente en el diagnóstico.

## Introducción

El diagnóstico del síndrome de Cushing (SC) (origen suprarrenal) sigue siendo un reto ya que sus síntomas y signos clínicos se superponen a los de otras enfermedades.

Dada su escasa frecuencia es necesario contar con un protocolo bien establecido que, de forma escalonada, permita diagnosticar a los pacientes con hipercortisolismo endógeno mediante datos clínicos y pruebas sencillas, para continuar posteriormente con exploraciones más complejas y específicas que permitan conocer su origen y tratamiento [1, 2].

Ninguna de las pruebas diagnósticas del SC tiene una sensibilidad superior al 90%, por lo que a veces se requieren procedimientos más invasivos como el cateterismo de senos venosos petrosos inferiores (CSPI) [3, 4].

Para el estudio inicial del SC, se debe determinar, en dos ocasiones, el cortisol libre urinario (CLU) en orina de 24 h, el cortisol sérico tras supresión nocturna con 1 mg de dexametasona, y/o el cortisol salival nocturno (CSN) a las 23 h [5]. Tras las pruebas de cribado, 2 resultados positivos de CLU serían diagnósticos.

El diagnóstico etiológico comprende la evaluación de la dependencia a adrenocorticotropina (ACTH) mediante la medición de su concentración [1], aquellos casos con valores >15 pg/mL serían orientativos de SC ACTH dependiente [5].

La medición del cortisol sérico tras la frenación con 8 mg de dexametasona (especificidad 67%, sensibilidad 79%) y el test de estimulación con corticoliberina (CRH) (especificidad 88–93%, sensibilidad 91–100%), son utilizados para diferenciar el origen hipofisario o ectópico (SCE) del SC ACTH dependiente. Las dosis altas de corticoides suprimen la secreción de ACTH en la mayoría de los tumores hipofisarios, mientras que no se inhibe en los tumores neuroendocrinos ectópicos [6]. La prueba de CRH se realiza inyectando 100 µg de CRH sintética i.v, en adultos, seguido de la extracción de muestras en distintos tiempos para la medición de cortisol y ACTH, considerando orientativo de Enfermedad de Cushing (EC) (origen hipofisario), un aumento de las concentraciones de ACTH >50% o de cortisol plasmático >20% respecto al valor basal.

La realización de resonancia magnética nuclear (RMN) hipofisaria facilita la localización del tumor, sin embargo, los resultados no siempre son concluyentes.

En los casos de resultados bioquímicos discordantes, o falta de evidencia de lesiones en la RMN, puede ser necesario realizar el CSPI. Éste está basado en el drenaje venoso de la hipófisis anterior al seno cavernoso y de este a senos petrosos inferiores (SPI). Tras la cateterización bilateral de los SPI, se mide ACTH basal y tras estímulo con CRH (a los 3, 5 y 10 min) en muestras obtenidas simultáneamente en senos petrosos y en vena periférica. Un resultado de gradiente de ACTH Central/Periférico (C/p) ≥2 en las muestras basales, o ≥3 en las muestras tras estímulo, son indicativos de EC. Un gradiente derecha/izquierda (o viceversa) ≥1,4 entre los dos SPI sugiere una localización del tumor en el lado del seno petroso con mayor concentración de ACTH [1, 2].

El caso que presentamos a continuación refleja el desafío diagnóstico de esta patología.

## Caso clínico

Presentamos el caso de un varón de 34 años remitido a Endocrinología por sospecha de SC tras presentar osteopenia/osteoporosis en densitometría ósea realizada por microfracturas metatarsianas de estrés, ganancia de peso, rubor e irritabilidad. No presentaba antecedentes clínicos personales ni familiares de interés. La exploración física reveló redondeamiento facial, ligera HTA, acúmulo de grasa retrocervical, sin estrías abdominales.

Los resultados del laboratorio, incluyendo la función hepática y renal, resultaron dentro del intervalo de referencia. Tras excluir la toma de esteroides exógenos, y ante la sospecha clínica de un hipercortisolismo endógeno, se realizaron las pruebas de cribado. Las concentraciones de cortisol sérico, salival, urinario (tras extracción con diclorometano) y de ACTH se midieron mediante una electroquimioluminiscencia (ECLIA) en un analizador Cobas (Roche Diagnóstics). Los resultados fueron los siguientes: CLU1 818 μg/24 h y CLU2 636,5 μg/24 h (VR: 10–120), frenación con 1 mg de dexametaxona 14,3 μg/dL (VR<1,8) y CSN 1,22 μg/dL (VR<0,27). Estos resultados confirmaron un SC endógeno. Las concentraciones de ACTH basal elevados (71 pg/mL (VR: 9–52)) para los niveles de cortisol, indicaban SC ACTH dependiente.

De las pruebas realizadas para identificar el origen de secreción de ACTH la supresión de cortisol con 8 mg de dexametasona sugería un origen hipofisario (de 21 μg/dL a 5,9 μg/dL, reducción >50%). Sin embargo, en la respuesta al test de CRH no se objetivó un incremento de ACTH >50% ni de cortisol >20%, considerado criterio de EC (Tabla 1) [5]. La RMN hipofisaria no evidenció la presencia de un adenoma.

**Tabla 1:** Resultados del test de CRH^a^.

Tiempo, min	Cortisol, µL/dL	Incremento de cortisol (>20%)	ACTH, pg/mL	Incremento de ACTH (>50%)
Basal	14,7	–	46	–
15′	16,4	11,56	46,2	0,44
30′	13,2	−10,20	39,7	−13,7
45′	11,6	−21,09	38,9	−15,43
60′	10,7	−27,21	34,3	−25,43
90′	10,1	−31,29	33,8	−26,52
120′	8,9	−39,46	38	−17,39

^a^Administración de 1 μg/kg de peso de Corticorelina por vía endovenosa y extracción de sangre en los tiempos indicados para la determinación de cortisol y ACTH (adrenocorticotropina).

Ante los resultados discordantes obtenidos, se realizó el CSPI (el paciente firmó el consentimiento informado). El gradiente ACTH (C/p) tras estímulo con CRH fue superior a 3 confirmando el origen hipofisario (Tabla 2). El gradiente interpretoso sugirió localización derecha del tumor.

**Tabla 2:** Resultados del CSPI^a^.

Tiempo, min	ACTH periférica, pg/mL	ACTH derecha, pg/mL	ACTH izquierda, pg/mL	Gradiente derecha/periférica	Gradiente izquierda/periférica	Inter-senos SPII/SPID	Inter-senos SPID/SPII
0′	29,93	35,31	37,59	1,17	1,25	1,06	0,93
3′	30,92	111,6	39,75	**3,60**	1,28	0,35	2,80
5′	34,13	189,9	181,1	**5,56**	**5,30**	0,95	1,04
10′	38,12	309,6	147,7	**8,12**	**3,87**	0,47	2,09

^a^Administración de 100 µg de Corticorelina por vía endovenosa y extracción de sangre de ambos senos petrosos inferiores (derecho e izquierdo) y de vena periférica para la determinación de ACTH a los 0, 3, 5 y 10 min. Valores por encima de la ratio.

El paciente fue intervenido mediante cirugía transesfenoidal. En el estudio anatomopatológico la inmunohistoquímica fue negativa para ACTH, sin embargo, la disponibilidad en nuestro centro de incluir el estudio del factor de transcripción Tpit, nos permitió identificar el origen corticotropo del tumor. El Tpit regula específicamente la expresión del gen proopiomelanocortina (*POMC*) en los tumores corticotropos [3]. Su inclusión nos ha permitido tipificar correctamente algunos subtipos de tumores hipofisarios que hubieran sido clasificados como null cell.

## Discusión

El diagnóstico del SC resulta sencillo cuando el cuadro clínico, las pruebas bioquímicas y de imagen son concordantes, permitiendo una correcta localización de la enfermedad. Sin embargo, en algunas ocasiones, como ocurre en nuestro caso, el SC ACTH-dependiente puede representar un reto diagnóstico.

Identificar la causa de un SC ACTH-dependiente es de crucial importancia ya que requerirá abordajes terapéuticos diferentes. En la mayoría de los pacientes con EC la exéresis quirúrgica del tumor es de primera elección [5].

Los algoritmos utilizados para la diferenciación entre EC y SCE generalmente se basan en los resultados de pruebas bioquímicas y estudios de imagen. Sin embargo, las diferencias bioquímicas entre ambas entidades con frecuencia no son significativas resultando compleja su diferenciación. En estos casos es de gran ayuda la realización de pruebas funcionales que proporcionen una mayor sensibilidad y especificidad diagnósticas.

Los resultados bioquímicos en nuestro paciente, permitieron realizar el diagnóstico inicial de SC, que tras medir concentraciones de ACTH, se identificó como ACTH dependiente.

El test de estimulación con CRH en sangre periférica permite identificar casos de SC hipofisario tras un incremento en las concentraciones séricas basales de ACTH igual o superior al 50%. Se fundamenta en el hecho de que los tumores hipofisarios corticotropos expresan fuertemente receptores de CRH y las moléculas de la vía de señalización y, por lo tanto, responden a este péptido liberando cantidades excesivas de ACTH, a diferencia de los tumores ectópicos [9]. En nuestro paciente la falta de respuesta al test de CRH sugería origen ectópico en la producción de ACTH, resultado que por otra parte, era discordante con el obtenido tras la frenación con 8 mg de dexametasona, a favor de un origen hipofisario.

En la práctica clínica, como queda reflejado en nuestro caso, ninguna de las pruebas actualmente disponibles muestra una precisión diagnóstica del 100%. La prueba de supresión con dosis altas de dexametasona es la principal prueba diagnóstica no invasiva. Sin embargo, la sensibilidad y la especificidad son sólo del 60% al 80% [7, 8].

Por otro lado, las pruebas de imagen empleadas para la localización de los tumores pueden resultar inconcluyentes. Hasta el 40% de los pacientes con enfermedad de Cushing tienen microadenomas ocultos no visibles en una RMN hipofisaria realizada en centros experimentados [9] y hasta un 10% de la población general pueden presentar incidentalomas [10], [11], [12]. Esto sería concordante con la falta de hallazgos significativos en la RMN realizada en nuestro paciente.

El CSPI es considerado el patrón de oro en el diagnóstico diferencial entre EC y SCE. Ofrece una elevada sensibilidad y especificidad para su distinción, puede ser de utilidad en la localización de lesiones hipofisarias, y presenta seguridad y tolerancia para el paciente [10]. Tradicionalmente, en esta prueba, se han establecido como puntos de corte gradiente SPI/periférico (basal (0′))>2 o gradiente SPI/periférico (estimulado)>3 [13–15] para el diagnóstico de EC. Estos umbrales son los utilizados en el caso expuesto. No obstante, es necesario tener en cuenta que estos puntos de corte pueden depender de los ensayos de ACTH y cortisol utilizados, además de estar condicionados por el limitado número de casos de SCE incluidos en las series de análisis del rendimiento diagnóstico de las pruebas [9].

En nuestro paciente los resultados obtenidos en el cateterismo (ACTH C/p>5 tras estímulo) fueron determinantes para llegar al diagnóstico de EC.

A pesar de tratarse de una excelente prueba diagnóstica el CSPI no se encuentra exento de limitaciones presentando falsos negativos y positivos Las causas más comunes de falsos negativos incluyen la existencia de SPI atróficos, colocación inadecuada de los catéteres, o un drenaje venoso anómalo [5]. Los falsos positivos podrían ser el resultado de una supresión incompleta del eje hipofisario en casos de SC suprarrenal o ectópico, o en pacientes con SC cíclico o leve [5].

El hematoma inguinal por acceso femoral es la complicación más común, observada en menos del 5% de los pacientes. En raras ocasiones, se han informado complicaciones más graves, como trombosis venosa profunda, embolia pulmonar o lesión cerebral [6] (hemorragias o infartos del tronco encefálico) [15].

## Conclusiones

El CSPI es una técnica con alta sensibilidad y especificidad en el procedimiento diagnóstico del SC ACTH-dependiente, cuando las pruebas complementarias de uso habitual no aportan certeza diagnóstica, sin suponer además elevados riesgos.
